# Virtual Reality in Chronic Conditions: An Umbrella Review

**DOI:** 10.3390/nursrep16020057

**Published:** 2026-02-10

**Authors:** Ilaria Marcomini, Giulia Villa, Laura Ingrande, Gaia Latini, Andrea Poliani, Duilio Fiorenzo Manara, Debora Rosa

**Affiliations:** 1 Center for Nursing Research and Innovation, Faculty of Medicine and Surgery, Vita-Salute San Raffaele University, 20132 Milan, Italy; marcomini.ilaria@unisr.it (I.M.); villa.giulia@hsr.it (G.V.); lauraingrande98@gmail.com (L.I.); latini.gaia@hsr.it (G.L.); manara.duilio@hsr.it (D.F.M.); rosa.debora@unisr.it (D.R.); 2 Department of Medical Oncology, IRCCS Ospedale San Raffaele, 20132 Milan, Italy

**Keywords:** chronic conditions, non-pharmacological interventions, rehabilitation, umbrella review, virtual reality

## Abstract

**Background.** Virtual reality (VR) is emerging as a non-pharmacological tool to support rehabilitation and self-management. Evidence of its effectiveness, however, remains fragmented. This umbrella review synthesized systematic reviews and meta-analyses on VR interventions in chronic conditions. **Methods.** Following the Joanna Briggs Institute Manual for Evidence Synthesis, comprehensive searches were conducted in MEDLINE, CINAHL, Cochrane Database, Web of Science, and Scopus. Eligible studies were systematic reviews and meta-analyses assessing VR interventions. Two reviewers independently performed screening, quality appraisal, and data extraction. **Results.** Seventeen reviews, including 229 primary studies, were analyzed. Stroke and chronic obstructive pulmonary disease were most frequently investigated. VR tools ranged from web- and smartphone-based systems to wearable devices and interactive games. Significant improvements were reported in respiratory outcomes, functional mobility, balance, and psychological symptoms. Cognitive effects were mixed. Reported adverse events, mainly cybersickness and dizziness, were mild. **Conclusions.** VR may improve physical, respiratory, psychological, and selected cognitive outcomes in chronic conditions. Despite study heterogeneity, evidence supports its integration into chronic care. Future work should standardize protocols, assess long-term effects, broaden target populations, and address equity and ethical issues to fully realize VR’s potential as a person-centered tool.

## 1. Introduction

According to the World Health Organization [1], chronic conditions are long-lasting, non-communicable health states characterized by a generally slow progression and a need for continuous management over time. They encompass not only chronic diseases but also a wide range of persistent neurological, musculoskeletal, mental, metabolic, and disabling conditions.

In recent years, virtual reality (VR) has emerged as a promising tool for managing chronic conditions. However, the definition of VR remains highly heterogeneous within the healthcare literature. A recent healthcare-focused systematic review identified more than 100 distinct definitions of VR, highlighting the lack of conceptual consensus and the variability in how VR-based interventions are described and operationalised across studies [2].

VR is increasingly applied in home, hospital, and outpatient care with robust evidence in rehabilitation. Compared to traditional exercise programs, VR-based activities have been shown to enhance motivation and adherence [3]. This is especially relevant in contexts where rehabilitation access is limited by high costs and shortages of trained therapists [4]. Beyond physical rehabilitation, VR has demonstrated benefits as a non-pharmacological intervention, such as reducing cancer-related fatigue [5] and providing immersive distraction techniques to alleviate psychological burden [6]. In respiratory care, VR combined with pulmonary rehabilitation has been shown to improve lung function and exercise performance in patients with COPD [7]. Additionally, VR shows promise in the field of clinical neurorehabilitation, as studies reveal statistically significant positive effects of VR-based therapies on executive function, memory, and visuospatial skills in stroke patients [8].

From a nursing perspective, VR is becoming increasingly important in daily clinical practice, where nurses often support patient education, guide rehabilitation activities, and implement non-pharmacological strategies for managing symptoms. VR-based interventions are already employed by nurses to decrease anxiety and pain during procedures [9], promote adherence to medications [10], and boost patient engagement in self-management programs for chronic conditions [11]. These uses closely align with the principles of person-centered and evidence-based nursing, highlighting the importance of understanding how VR can be integrated into multidisciplinary chronic care pathways.

Given the rapid expansion of VR applications in chronic conditions management, there is a pressing need to systematically synthesize evidence across conditions, outcomes, and care settings. In this review, chronic conditions were conceptualized based on shared long-term care, rehabilitation, and self-management needs, rather than disease-specific characteristics. This perspective reflects the common involvement of prolonged interventions, patient engagement, and functional recovery processes targeted by VR across different conditions. Accordingly, this umbrella review was designed to identify, evaluate, and analyze systematic reviews and meta-analyses on the clinical effects of VR in patients with chronic conditions, thereby providing a comprehensive evidence base for researchers, healthcare providers, and policymakers.

## 2. Materials and Methods

### 2.1. Aim

The review question addressed was: “How is virtual reality used to support the management of chronic conditions?”.

This review has been conducted in accordance with the Joanna Briggs Institute Manual for Evidence Synthesis [12].

### 2.2. Conceptualisation of Virtual Reality

In light of the substantial heterogeneity in how virtual reality is defined in the healthcare literature [2] and considering the scope and objectives of an umbrella review, the present study adopted a broad and inclusive conceptualization of VR. Specifically, VR was defined according to McCloy [13] as “a collection of technologies that allow people to interact efficiently with three-dimensional computerized databases in real time using their natural senses and skills.” This technology-agnostic definition emphasizes real-time interaction and multisensory engagement rather than specific devices or degrees of immersion.

### 2.3. Search Strategy

The research question was structured using the PICO Framework (Participants/Intervention, Comparison, Outcomes) to guide eligibility criteria, keywords, and search strategies [12].

Following the JBI three-step approach [12], an initial limited search in MEDLINE was performed to identify relevant reviews and analyze key text words and index terms, including “systematic review” and “meta-analysis.” Comprehensive searches were then conducted in MEDLINE via PubMed, CINAHL, Cochrane Database, Web of Science, and Scopus using terms associated with the research topic combined with Boolean operators. Database-specific filters were applied, and the full search strategies are reported in Supplementary Materials (Table S1). Finally, the reference lists of all included reviews were screened for additional studies.

### 2.4. Eligibility Criteria

#### 2.4.1. Inclusion Criteria

Study selection was based on addressing the research question and the PICO Framework (Table 1) [12]. Systematic reviews and meta-analyses on the use of virtual reality in the management of chronic conditions were included, with no restrictions on date, geographic location, or language.

#### 2.4.2. Exclusion Criteria

In accordance with the predefined eligibility criteria, we excluded studies whose focus did not align with the scope of the review. Specifically, studies addressing caregiver-related outcomes, acute disease conditions, and pharmacological interventions. Furthermore, studies that did not adopt a systematic review or meta-analytical methodology were excluded to maintain consistency and ensure the methodological robustness of the evidence synthesized. We also excluded reviews focusing exclusively on anxiety or depression, as these conditions are not classified as chronic diseases under the DSM-5 criteria [14].

### 2.5. Study Selection

The selection of studies was performed in accordance with the eligibility criteria. Study selection was conducted in two distinct stages: (1) title and abstract screening, and (2) full-text screening. At both stages, two reviewers (L.I. and G.L.) independently screened all retrieved citations against the eligibility criteria. Any disagreements between the two reviewers were discussed and resolved through consensus; when consensus could not be reached, a third reviewer (I.M.) was consulted. The process and decisions taken at each stage were documented in detail to ensure transparency and reproducibility, as recommended by the JBI Manual for Evidence Synthesis [12]. The entire screening process was performed using the Rayyan web application [15].

### 2.6. Assessment of Methodological Quality of Included Studies and Quality of Evidence

Two reviewers (L.I., G.L.) independently appraised quality using the JBI Critical Appraisal Tool [16]. The tool comprises 11 items rated as “Yes,” “No,” “Unclear,” or “Not applicable.” Any disagreements were resolved through discussion with a third reviewer (I.M.).

For descriptive and interpretative purposes, reviews were categorized into three levels of methodological quality (high, moderate, and lower quality) based on the number and pattern of affirmative (“Yes”) responses across the 11 JBI items. Reviews were considered high quality when most criteria were met (generally 9–11 “Yes” responses) with no major methodological concerns; moderate quality when several criteria were met (typically 6–8 “Yes” responses) but some limitations were identified; and lower quality when fewer criteria were satisfied (≤5 “Yes” responses) or when multiple critical items were rated as “No” or “Unclear”.

The certainty of the evidence was evaluated using the Grading of Recommendations, Assessment, Development and Evaluation (GRADE) approach [17]. When available, GRADE ratings reported in the included systematic reviews were extracted and summarized by outcome. Evidence was classified into four levels (high, moderate, low, or very low) based on the overall confidence in the effect estimates, in order to inform the strength of the resulting recommendations. When GRADE assessments were not reported, no formal reclassification was conducted; instead, the confidence in the evidence was discussed narratively, drawing on methodological quality, sample size, consistency of findings, and heterogeneity.

### 2.7. Data Collection

Data were extracted using the standardized JBI Data Extraction Tool for Systematic Reviews and Research Syntheses [12]. Extracted information included specific items such as data collected on patients’ chronic conditions, age, sex, type of device used, intervention details, study location, publication year, disease duration, and key findings. Only data reported in the included reviews were used.

### 2.8. Overlap of Primary Studies

To assess the degree of overlap of primary studies across the included systematic reviews, a citation matrix was constructed [17]. Only primary studies appearing in at least two reviews were retained in a reduced citation matrix to improve readability and to identify the main sources of overlap.

The degree of overlap was quantified using the Corrected Covered Area (CCA), calculated according to the method proposed by Kirvalidze et al. [18]. The total number of primary study occurrences across reviews (*N*), the number of unique primary studies (*n*), and the number of included systematic reviews (*r*) were used to compute the CCA using the following formula:

$$\mathrm{CCA}=\frac{N-n}{n\times \left(r-1\right)}$$

To facilitate interpretation, CCA values were visually summarized using the GROOVE (Graphical Representation of Overlap of OVErviews) approach [19], in which different colors were assigned to predefined CCA ranges to indicate increasing levels of overlap among reviews. Specifically, green indicated no overlap (CCA = 0), yellow slight overlap (0–5%), orange moderate overlap (6–10%), light brown high overlap (11–15%), and dark brown very high overlap (>15%).

## 3. Results

### 3.1. Study Screening and Selection

**Figure 1:**
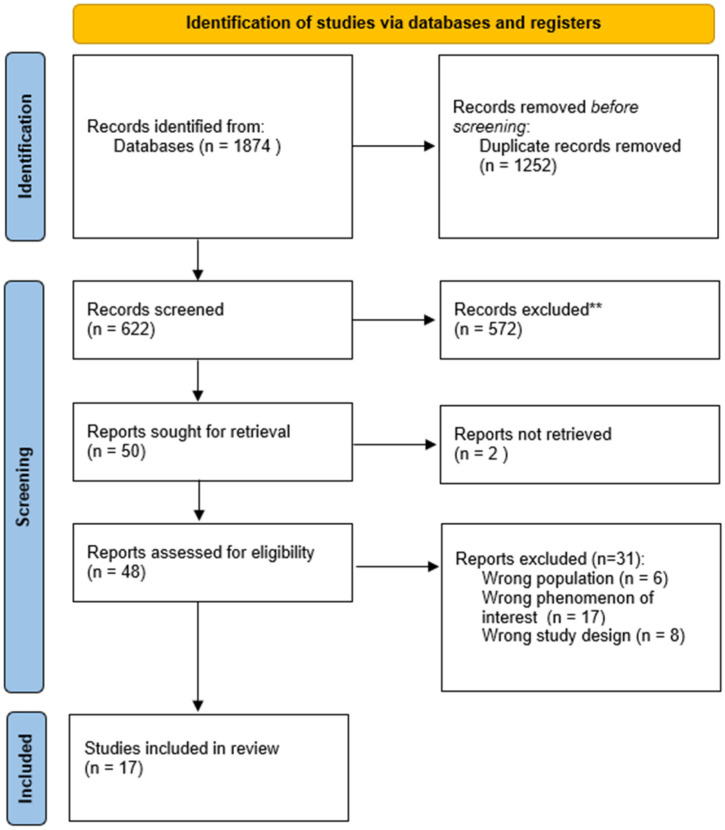
PRISMA Flow Diagram 2020—Selection process. ** Records excluded during title and abstract screening.

A total of 1874 records were identified from various databases. After removing duplicates, 1252 records potentially meeting the inclusion criteria were selected. The selection process was performed using the Rayyan web application [15] and illustrated with the PRISMA flow diagram [20] (Figure 1).

572 articles were excluded in the title-abstract selection, leaving 50 full-text articles, of which 2 lacked full-text availability. The main reasons for full-text exclusion were: diverse populations, variations in phenomena of interest, and inappropriate study designs. The full-text selection involved a total of 17 reviews.

### 3.2. Study Characteristics

The 17 reviews included were published between 2014 [21] and 2024 [22] in various countries. China accounted for 29.41% of the total reviews [7,8,23,24,25], followed by Brazil with 11.76% [21,26]. Other countries represented include Canada [27], Greece [28], Spain [29], Italy [30], Saudi Arabia [22], Great Britain [31], Jordan [6], Taiwan [32], Korea [33], and Denmark [34] each contributing 5.88% of the total.

The majority of the reviews (88%) are meta-analyses [7,8,21,22,23,24,25,27,28,29,32,33,34], while the remaining 12% consist of systematic reviews [6,30,31].

The number of primary studies in the included reviews ranged from five to 31, resulting in a total of 229 primary studies.

Most of the included studies were randomized controlled trials, comprising 88.29% [6,7,8,21,22,23,24,25,26,27,28,29,30,31,32,33,34]. A smaller percentage were observational studies (9%) [31], followed by systematic reviews (1.80%) [6], secondary analyses of randomized controlled trials and open-label trials (0.90%) [30].

Across the included reviews, several studies listed at least one author affiliated with a school, department, or faculty of nursing [6,8,23,25,29,34,35].

**Figure 2:**
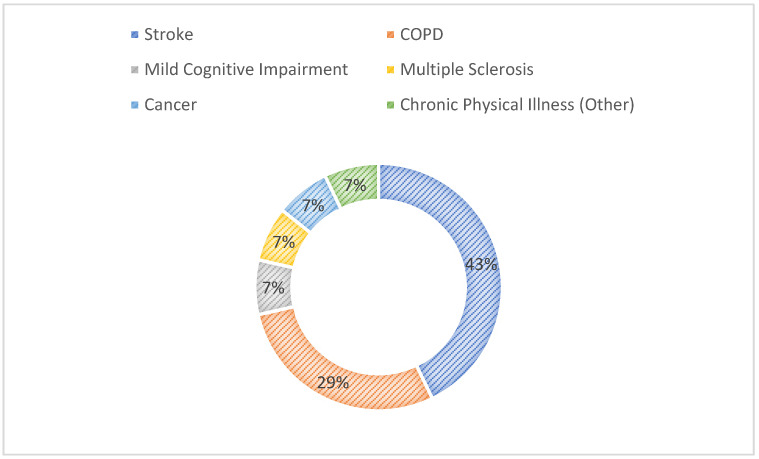
Conditions considered in the included studies.

The most reported chronic condition was stroke (43%) [8,21,22,24,27,33], followed by COPD (29%) [23,28,29,35] (Figure 2).

Sample sizes across the included studies varied considerably, ranging from 154 [21] to 1257 participants [31]. Among the studies that reported gender-specific data, the number of participants ranged from 154 [22] to 793 [31] for women and 189 [29] to 464 [31] for men. One study did not report its sample size [34].

The implementation of VR interventions varied along the continuum of patient care. VR was applied at different stages of care, including the acute, post-acute, and chronic phases of treatment [6,7,8,21,23,25,26,27,28,29,30,31,32,33,34].

### 3.3. Assessment of Methodological Quality of Included Studies

Most studies met the key criteria, particularly in terms of search strategy, inclusion, data extraction, and synthesis (Table 2). Common gaps included unclear research questions (Q1), a lack of independent appraisal reporting (Q6), and inconsistent assessment of publication bias (Q9). Reviews were classified into three methodological quality categories based on the JBI critical appraisal results. High-quality reviews were those meeting most JBI criteria (typically 9–11 “Yes” responses) and accounted for approximately one-third of the included reviews [6,8,24,26,30,31]. These reviews showed no major methodological concerns.

The majority of reviews were classified as moderate quality, fulfilling several key methodological criteria but presenting some limitations, particularly regarding protocol registration, assessment of publication bias, and clarity of search strategy reporting [22,23,25,28,29,32,33,34,35].

Finally, a small proportion of reviews showed lower methodological quality [21,27]. These reviews showed a higher number of “No” or “Unclear” ratings across multiple JBI items, particularly in domains related to the assessment of publication bias and transparency of methodological procedures.

### 3.4. Overlap of Primary Studies

As reported in Table 3, according to the GROOVE classification, the CCA matrix shows that most pairwise comparisons fall in the Green category (CCA = 0), indicating no overlap between reviews. This pattern is evident for reviews addressing distinct clinical conditions, such as multiple sclerosis (Rev 5) [26], mixed chronic conditions (Rev 8) [31], cancer-related fatigue (Rev 10) [6], and chronic musculoskeletal disorders (Rev 12) [32], supporting a high level of independence of the evidence base.

Slight overlap (Yellow, 0–5%) is observed mainly among reviews focused on COPD, including Rev 1 [28], Rev 2 [29], Rev 3 [23], and Rev 11 [7], reflecting partial sharing of randomized controlled trials within a relatively well-defined clinical area.

Moderate overlap (Orange, 6–10%) emerges primarily in comparisons involving stroke-related reviews, such as Rev 6 [24], Rev 9 [8], and Rev 11 [7], indicating convergence on common intervention paradigms and outcome measures in post-stroke rehabilitation.

Higher levels of overlap are concentrated within the chronic stroke and neurological rehabilitation cluster, including Rev 13 [33], Rev 14 [34], Rev 15 [30], Rev 16 [21], and Rev 17 [27]. In this group, high overlap (Light Brown, 11–15%) and very high overlap (Dark Brown, >15%) are observed, with the highest CCA values found between Rev 13–Rev 14 (23.5%), Rev 14–Rev 15 (22.2%), and Rev 14–Rev 17 (21.4%). This reflects substantial redundancy of primary studies, likely due to shared focus on chronic stroke populations and repeated inclusion of landmark RCTs.

### 3.5. Virtual Reality Tools

Across the included systematic reviews, VR interventions were delivered through a remarkably heterogeneous spectrum of technologies, ranging from commercial exergaming devices to fully immersive head-mounted display (HMD) systems and bespoke rehabilitation platforms. In pulmonary rehabilitation, several studies implemented commercial systems such as the Wii Fit, which enables interaction with virtual environments through a handheld controller and, in some cases, the Wii Balance Board to support balance-oriented tasks [7,23]. The Nintendo Wii console, more broadly, was also employed to simulate functional activities and whole-body movements using motion-sensitive controls [22,24,25,26]. Another widely used system was the Xbox Kinect, a motion-capture device capable of recognizing the user’s outline and body segments to provide real-time biofeedback during stepping, reaching, or multidirectional walking tasks [23,25,26].

More immersive pulmonary rehabilitation protocols relied on specialized systems such as the BioMaster virtual situational interactive training system, which generates realistic interactive simulations through combined hardware and software interfaces to reproduce daily environments and promote functional breathing exercises [7,23]. Similarly, VR TierOne delivered strong visual, auditory, and kinesthetic stimulation through a head-mounted display, often using applications such as the Virtual Therapeutic Garden, an immersive metaphor in which environmental flourishing across sessions symbolizes clinical improvement and psychological engagement [23,28]. Additional COPD-focused interventions included somatosensory game-based platforms designed to stimulate pulmonary effort and whole-body movement [23]. Semi-immersive tools such as data gloves and simulated bicycles were also used to enhance perceptual involvement and motor compliance during therapeutic tasks [28]. This pattern of combining accessible non-immersive exergaming platforms with more advanced VR tools was also evident in COPD-focused interventions, where non-immersive VR (niVR) and immersive VR (iVR) systems were applied within the same therapeutic context [29].

In cognitive and neurological rehabilitation, VR interventions ranged from non-immersive exergaming systems to fully immersive HMD-based cognitive training. Programs targeting mild cognitive impairment employed Kinect- or Wii-based games simulating activities such as kayaking, as well as BioRescue, a pressure-sensitive platform configured to deliver visual feedback for postural control and cognitive–motor exercises [25]. Cognitive training often included modules delivered through head-mounted displays, where users completed daily life tasks, memory sequences, or executive-functioning exercises in virtual settings [25]. In multiple sclerosis rehabilitation, VR sessions typically began with a warm-up on a stationary bicycle, followed by Wii Fit balance games or Xbox 360 Kinect balance exercises involving multidirectional steps, unilateral or bilateral postural tasks, and dynamic weight-shifting [26]. Cycling-based systems such as Speed-Interactive Pedaling Training (SIPT) combined stationary bikes with the Virtual Active smartphone application, which projected immersive environments, such as mountain trails or urban landscapes, whose movement synchronized with the patient’s pedaling rhythm [22]. Driving-Based Interactive Video Games (DBIVG) introduced complex motor-cognitive tasks through steering wheels, pedals, and cockpit setups simulating car-racing environments demanding continuous visuomotor engagement [22].

Stroke rehabilitation deployed a broad range of VR modalities, including the Wii Balance Board for posture and weight-shift training [24,27] and Kinect-based exergaming through titles such as Kinect Sports, Kinect Adventures, and Your Shape Fitness Evolved to promote lower-limb coordination, balance, and motor performance [24]. Some interventions integrated VR with conventional physiotherapy techniques including stretching, gait training, joint mobilization, strengthening, cycling ergometry, stair climbing, and task-specific functional exercises such as door unlocking, cooking, bathing, or tool-use simulations, all performed within virtual task environments [24]. More technologically advanced approaches used treadmill-based virtual environments where real-world video recordings or augmented simulations were synchronized with treadmill speed to improve gait stability, obstacle negotiation, and locomotor adaptability [21,27]. Other stroke protocols incorporated balance games, aerobic activities, muscle-strengthening routines, or bilateral upper-limb rehabilitation delivered through Wii Sports & Resort, Wii Play Motion, Let’s Tap, Xbox Kinect sports titles, and mechanically supported VR-assisted reaching and grasping exercises [33].

In psychological and well-being interventions, immersive VR systems were prevalent, leveraging head-mounted displays such as Vuzix Wrap, ezVision, Zeiss VR One, VR Box 2, and Oculus Quest to deliver calming natural landscapes or emotionally engaging environments [31]. Virtual scenarios included environments such as Ocean Rift (underwater exploration), Happy Place (tranquil nature-based scenes), Second Life (exploratory virtual worlds), and Nature Treks (ambient naturalistic environments), as well as unspecified platforms projecting scenes such as forests, beaches, safaris, and meditative settings [31]. Some programs also employed VR-based mindfulness, VR-CALM environments, and 360° immersive videos to alleviate cancer-related fatigue, anxiety, and emotional distress [6]. Studies also included theBlu for underwater relaxation, Google Earth VR for virtual travel, Unreal Engine–based guided relaxation scenarios, and personalized reminiscence VR videos filmed with 360° cameras [31].

A number of reviews described mixed or unspecified VR systems. Some employed combinations of immersive and non-immersive interactive VR without specifying the exact devices [32]. Others used general screen-based or head-mounted VR without reporting the underlying hardware [8]. Finally, several studies involving balance rehabilitation, motor-cognitive dual-task training, or older adult motor interventions referenced VR use but did not provide sufficient technological detail [30,33,34].

The virtual reality intervention was implemented with varying temporal frequencies, ranging from a minimum of 10 min once daily [6] for a fortnight to a maximum of 120 min five times weekly for four weeks [33].

### 3.6. Outcomes

The outcomes assessed across the included studies were organized into five main macro-categories and each category was subdivided into specific thematic subdomains to ensure conceptual clarity and consistency in outcome classification: (a) Movement, Balance, and Motor/Functional Skills, (b) Quality of Life, (c) Cognitive and Memory Outcomes, (d) Psychological, Emotional, and Pain Outcomes, and (e) Adverse Events and Safety Outcomes.

#### 3.6.1. Movement, Balance, and Motor/Functional Skills

This outcome category comprises physical mobility, motor function, postural control, and overall performance. A first focus was motor and muscular function, primarily assessed through gait and endurance tests. The 6-Minute Walk Distance (6MWD) [7,23,27,28,34] measured sustained effort, while short-distance protocols such as the 10-Meter, 1-Minute, and 3-Minute Walk Tests [27,34], captured mobility in restricted timeframes. The Timed Stair Test (TST) [27] added task-specific insights into lower-limb strength and step negotiation. Collectively, these tools evaluated walking endurance, functional mobility, and fatigue resistance.

A second area concerned balance and postural control, examined with both static and dynamic measures. The Berg Balance Scale (BBS) [24,26,27] and Brunel Balance Assessment (BBA) [27] assessed stability in varied conditions. Transitional balance was evaluated through the Timed Up and Go (TUG) [24,26,27,34] while reach-based tools such as the Functional Reach Test and Anterior Reach Test (ART) [24,27] quantified standing stability. Broader mobility and fall risk were examined via the Tinetti Performance-Oriented Mobility Assessment [27,30] and the 30-Second Sit to Stand Test (30-SSTS) [27]. Complementary biomechanical parameters (center of pressure, stability index, postural sway) provided fine-grained monitoring, especially under dual-task or cognitively demanding conditions [30].

Finally, range of motion and functional capacity were evaluated to capture flexibility, endurance, and fatigue. Joint mobility was measured with standardized protocols [33], while physical tolerance was tested with endurance-based measures [7,28]. Fatigue and exertional decline were quantified through performance-based protocols [6,26,31]. In addition, perceived exertional breathlessness was monitored with the Modified Medical Research Council Dyspnea Scale (mMRC) [23,28], linking mobility outcomes to respiratory effort.

#### 3.6.2. Quality of Life (QoL)

This outcome category addresses perceived quality of life, autonomy in daily living, and subjective well-being, explicitly excluding motor-related outcomes to distinguish functional independence from physical performance.

A central focus was the evaluation of Activities of Daily Living (ADL) and Instrumental Activities of Daily Living (IADL), which capture the ability to manage both basic self-care (e.g., bathing, dressing, feeding) and more complex tasks essential for independent living (e.g., shopping, transportation). These were frequently assessed using validated scales, particularly the Modified Barthel Index [24,25,33].

A second subdomain was perceived fatigue and vitality, reflecting subjective levels of energy, stamina, and daily tiredness. These measures are key to understanding how individuals experience resilience and endurance in everyday contexts [6,26,31].

Finally, overall quality of life was assessed through standardized instruments evaluating general well-being and health-related quality of life from the patient’s perspective [8,23,26].

#### 3.6.3. Cognitive and Memory Outcomes

This outcome category encompasses cognitive functioning and mental abilities, structured according to the domains assessed.

The first subdomain is global cognitive function, referring to overall cognitive performance. It was commonly evaluated using composite scores or broad cognitive assessment tools that provide a general estimate of cognitive status [8,23].

The second subdomain targets specific cognitive domains, offering a more detailed perspective. Memory (immediate and delayed recall) was assessed through dedicated tasks [8,25] often alongside attention, which was measured using similar instruments [8,25].

Executive function, encompassing planning, problem-solving, and cognitive flexibility, represented another key area, evaluated through validated cognitive tests [8,25,30]. Further domains included verbal fluency, visuospatial ability [8], and time perception considered within cognitive processes [6].

#### 3.6.4. Psychological, Emotional, and Pain Outcomes

This outcome category covers emotional well-being, psychological status, and pain perception, integrating both clinically significant conditions and broader affective experiences relevant to overall health.

The first subdomain, emotional and psychological state, was assessed through standardized tools measuring mood and mental health symptoms. Core instruments included the Beck Depression Inventory (BDI) [8,23], for depressive severity, and the Hospital Anxiety and Depression Scale (HADS), which evaluates anxiety (HADS-A) and depression (HADS-D) [23,28]. The State-Trait Anxiety Inventory (STAI) [23,31] was frequently applied to distinguish between transient and persistent anxiety states. Broader measures encompassed mood, psychological distress [31], and motivational or emotional responses related to healthcare experiences [34].

The second subdomain, pain, focused on patient-reported intensity and impact on daily life. The Visual Analogue Scale (VAS) was the most widely adopted tool, complemented by additional validated measures to capture the subjective experience of pain [6,31,32,34].

#### 3.6.5. Adverse Events and Safety Outcomes

Adverse events were systematically monitored in several reviews and included general treatment-related adverse events [23] and VR-specific side effects such as cybersickness, fall injuries, dizziness, and eye strain [34].

Stroke rehabilitation studies also incorporated stability metrics linked to fall risk [27], while motor-cognitive dual-task training monitored falls and instability episodes [30].

**Figure 3:**
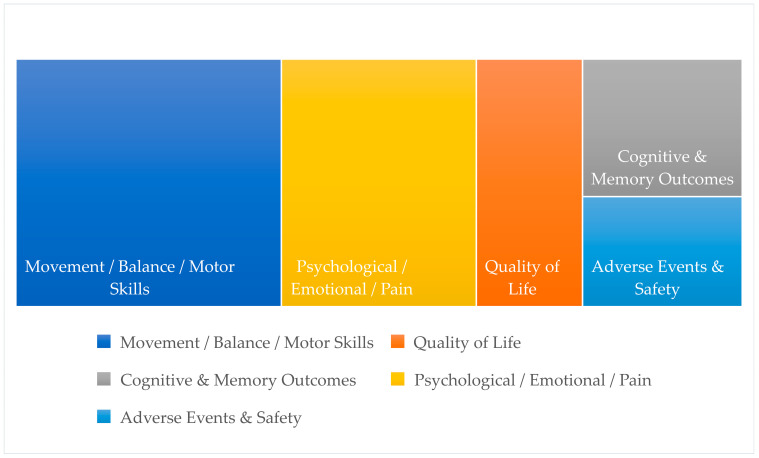
Distribution of outcome categories assessed across the 17 included reviews.

Figure 3 illustrates the distribution of outcome categories assessed across the 17 included reviews.

### 3.7. Efficacy of VR

Evidence from multiple meta-analyses indicates that VR-based pulmonary rehabilitation is effective on selected respiratory and functional outcomes in patients with COPD. Chai et al. [23] reported significant improvements in FEV1% predicted (*p* = 0.007), FEV1/FVC (*p* < 0.001), dyspnea (*p* < 0.001), and symptoms of depression (*p* = 0.033) and anxiety (*p* = 0.036), although no significant effect was observed for absolute FEV1 (*p* = 0.124). Similarly, Patsaki et al. [28] found a statistically significant improvement in pulmonary function (*p* = 0.002), while gains in exercise capacity were marginal and at the threshold of statistical significance (*p* = 0.05).

More recent evidence from Obrero-Gaitán et al. [29] showed significant improvements in functional capacity (*p* = 0.017) and functional mobility (*p* < 0.001), whereas effects on pulmonary function (FEV1) were small and supported by very low-certainty evidence (*p* = 0.048). In contrast, Liu et al. [7] reported no significant effect of VR on FEV1 (*p* = 0.38), although improvements were observed for FEV1/FVC and exercise capacity when VR was combined with conventional pulmonary rehabilitation. In individuals with mild cognitive impairment, Zhong et al. [25] demonstrated a statistically significant improvement in global cognitive function measured by MoCA (*p* = 0.03), supported by moderate-certainty evidence, whereas no significant effects were found when cognition was assessed with the MMSE (*p* = 0.61). Executive function improved significantly (*p* < 0.001), while no consistent effects were observed for delayed or immediate memory, attention, or instrumental activities of daily living (*p* > 0.05).

In contrast, in stroke populations, evidence was more heterogeneous. Zhang et al. [8] found no significant effect of VR on global cognition (*p* = 0.41) or attention (*p* = 0.58), but reported significant improvements in executive function (*p* = 0.03), memory (*p* = 0.02), and visuospatial ability (*p* = 0.006). Strong and consistent effects of VR were observed for balance and functional mobility in neurological populations. Shen et al. [24] reported significant improvements in balance (*p* < 0.00001), functional mobility (*p* < 0.0001), functional reach (*p* < 0.001), and ADL (*p* = 0.004) in stroke patients. These findings were corroborated by Iruthayarajah et al. [27] and Høeg et al. [34], who showed significant effects on BBS and TUG scores (*p* ≤ 0.001), while effects on walking endurance were inconsistent (*p* = 0.06).

Similarly, Rodrigues-Baroni et al. [21] demonstrated a significant increase in walking speed following VR-based gait training. In chronic musculoskeletal disorders, Kantha et al. [32] provided good-quality evidence that interactive VR significantly reduced pain compared with no rehabilitation and conventional rehabilitation, while effects on functional disability were not significant.

In cancer-related conditions, Alhusamiah et al. [6] and McGhee et al. [31] reported reductions in fatigue, pain, anxiety, depression, distress, and systolic blood pressure, supporting the feasibility and potential effectiveness of immersive VR, although statistical estimates were often derived from small or heterogeneous studies.

Across multiple sclerosis and mixed chronic conditions, Nascimento et al. [26] found no significant improvement in functional mobility, but significant benefits for fatigue, balance, and quality of life.

### 3.8. Quality of Evidence

Among the 17 included reviews, only two [25,29] explicitly applied the GRADE framework to evaluate the certainty of evidence, focusing on populations with mild cognitive impairment and COPD, respectively. For transparency and methodological rigor, only formally reported GRADE ratings from these reviews are presented. Table 4 summarizes the outcomes, pooled effect sizes, and GRADE certainty ratings for the two reviews.

For the remaining reviews, the certainty of the evidence was interpreted narratively, drawing on indicators of methodological rigor (PEDro, Cochrane RoB/RoB2, AMSTAR 2, ROBIS, EPHPP), sample size and statistical power, consistency of effects across studies, and reported heterogeneity, and subsequently mapped onto the prespecified outcome framework.

For Movement, Balance, and Motor/Functional Skills, most evidence derives from rehabilitation contexts involving different populations [7,21,22,23,24,26,27,28,29,30,33,34]. Most included primary studies were RCTs of moderate-to-good methodological quality and consistently demonstrated benefits in gait and endurance, balance and postural control, trunk stability, and functional mobility. Taken together, these findings support moderate confidence that VR contributes to improvements in motor- and balance-related outcomes, although the lack of blinding and variability in interventions limit stronger certainty.

For Quality of Life (QoL), including ADL, IADL, and perceived vitality, effects were generally smaller and more heterogeneous. Reviews across different rehabilitation settings [6,8,23,24,25,26,31,33] reported modest improvements in functional independence, fatigue, and health-related QoL; however, these findings were often based on a limited number of studies and heterogeneous assessment tools. Accordingly, confidence in VR-related QoL benefits can be considered low-to-moderate.

For Cognitive and Memory Outcomes, evidence is strongest in populations undergoing cognitive-oriented rehabilitation. Findings from several studies [8,23,25,30], indicate improvements particularly in executive and visuospatial functions, whereas effects on global cognition, memory, and attention are smaller and less consistent. Given the moderate methodological quality but substantial heterogeneity in cognitive tasks and interventions, there is moderate confidence for domain-specific cognitive gains (especially executive/visuospatial), and low-to-moderate confidence for global cognition and memory.

For Psychological, Emotional, and Pain Outcomes, Kantha et al. [32] provides comparatively robust RCT-based evidence supporting reductions in pain and disability, suggesting moderate confidence in this area. In other immersive VR applications [6,31], benefits have been reported in reducing anxiety, distress, and procedural pain; however, these findings are largely derived from small, heterogeneous, and often pilot studies, resulting in low-to-moderate confidence overall for mood and anxiety-related outcomes.

Finally, Adverse Events and Safety Outcomes were less consistently reported. Where explicitly assessed [23,27,30,31,34], VR interventions were generally well tolerated, with predominantly mild and transient effects (e.g., cybersickness, dizziness) and no consistent evidence of serious harm or increased fall risk. Nonetheless, due to incomplete and non-standardized reporting, certainty regarding safety remains low, although available data suggest a favorable safety profile when VR is delivered under appropriate clinical supervision.

## 4. Discussion

This umbrella review aimed to evaluate the effectiveness of VR interventions in chronic conditions, representing the first synthesis of its kind. Findings provide an evidence base for clinicians, guidance for educators on target populations and outcomes, and insights for policymakers and administrators to improve care delivery and strategic planning. Seventeen reviews published between 2014 and 2025 were included, reflecting a growing interest in VR applications in clinical contexts over the last decade [36]. Interestingly, the majority of the included reviews originated from China, a trend that may be attributed to greater technological investment and infrastructure in the healthcare sector. While this highlights China’s leading role in the development and adoption of digital health tools, it also raises important concerns regarding the generalizability of these findings to other settings, particularly low-resource environments, where accessibility and usability remain insufficiently addressed [37].

Methodological quality across the included reviews was generally acceptable, with a predominance of systematic reviews and meta-analyses of randomized controlled trials. Nevertheless, several reviews lacked clearly formulated research questions or independent quality appraisal, which may limit the robustness of their conclusions.

The clinical populations examined in the included studies were heterogeneous, with stroke and COPD emerging as the most frequently investigated conditions. This emphasis likely reflects the pronounced motor and respiratory impairments associated with these diseases, which align well with the rehabilitative potential of VR technologies [38,39]. In contrast, neurological and complex chronic conditions were significantly underrepresented. Given the increasing attention toward personalized, integrated, and long-term care in these populations, this underrepresentation constitutes a meaningful gap in the current body of evidence.

Importantly, this clinical imbalance is mirrored by methodological patterns in the evidence synthesis. The GROOVE-based analysis of the CCA demonstrated that overlap was not uniformly distributed across the evidence base but concentrated within specific pathology-driven clusters, particularly in chronic stroke and neurological rehabilitation. In these clusters, high to very high overlap suggests that conclusions may be influenced by a shared core of primary randomized controlled trials.

Another key finding is that VR interventions were mostly applied in the post-acute rehabilitation phase, likely due to greater feasibility and lower risks in medically stable patients. In contrast, the acute phase remains largely unexplored, representing a potential area for future research and clinical application [40].

VR interventions included web-based apps, smartphone systems, wearables, and gamified immersive tools aimed at enhancing engagement and motivation. However, the lack of a standardized definition of VR, together with wide variability in session duration and frequency, limits comparability and complicates clinical implementation. The development of such interventions would benefit from established theoretical frameworks [41], such as the Consolidated Framework for Implementation Research (CFIR) [42] or the Non-adoption, Abandonment, Scale-up, Spread, and Sustainability (NASSS) framework [43], which offer practical tools for addressing the complexity of real-world adoption across different healthcare settings.

Findings from the review showed that VR-based interventions led to clinically and statistically significant improvements across multiple outcome areas. However, while gains were seen in overall cognitive function and in executive functioning, especially on tasks that require cognitive flexibility and processing speed [8,25], the effects on delayed memory were minimal and did not consistently reach statistical significance. No significant effects were found for attention or verbal fluency [8]. These findings indicate that VR may be more effective for complex cognitive functions that are actively engaged during immersive and goal-directed tasks, while more basic or passive cognitive domains might need different or supplementary types of intervention [44]. For other outcome domains, including motor and balance-related skills, quality of life, safety and psychological outcomes, benefits were generally reported across reviews but were predominantly supported by narratively assessed evidence of variable methodological quality, resulting in low-to-moderate confidence in the robustness and generalizability of these effects.

### 4.1. Implications for Nursing Research and Practice

The results of this umbrella review suggest several important implications for nursing practice and research. Notably, several of the included reviews featured authors affiliated with schools or departments of nursing, underscoring the growing disciplinary engagement in evaluating and applying VR technologies within nursing-led models of care. From a practical perspective, nurses can use the findings of this synthesis to better understand how VR has been applied, the types of outcomes achieved, and the technological tools most commonly employed. This knowledge may inform clinical decision-making and guide the integration of VR-based interventions into patient education pathways. The demonstrated benefits in physical, cognitive, and psychological outcomes indicate that VR could be complementary to traditional nursing interventions aimed at promoting functional recovery, emotional well-being, and engagement in self-management.

From a research perspective, this review highlights several gaps that require further investigation. Although the growing body of evidence on VR in chronic conditions offers promising opportunities to integrate technology into patient-centered education and care, most studies have focused on post-acute phases and on conditions such as stroke and COPD. Further research in nursing is necessary to expand VR applications to other chronic and neurological populations and to assess its feasibility and safety in acute care settings. The heterogeneity of intervention designs and outcome measures also underscores the need to contribute to the development of standardized protocols and theoretical frameworks for implementation. Moreover, the long-term effects of VR interventions are still poorly understood due to a general lack of follow-up assessments. It is plausible that some of the observed outcomes may diminish over time without ongoing engagement strategies or adaptive protocols. Finally, the ethical, psychological, and social implications of VR in vulnerable populations are rarely addressed, limiting the current understanding of its broader impact.

### 4.2. Limitations

The limitations identified in this umbrella review largely reflect the characteristics of the reviews includes. A key challenge was the marked heterogeneity in terms of study populations, types of VR interventions, session duration, frequency, outcome measures and comparators. A major challenge was the substantial heterogeneity across reviews in terms of study populations, types of VR interventions, session duration, frequency, outcome measures, and comparators. In particular, comparators differed markedly between reviews and included usual care, conventional rehabilitation, alternative active interventions, or no intervention. As a result, the reported effect sizes and *p*-values are inherently relative to different reference conditions rather than representing absolute estimates of intervention effectiveness. This heterogeneity limits direct comparability across reviews, complicates interpretation of the synthesized findings, and reduces their generalizability across chronic conditions. In addition, although overlap was formally assessed using the CCA and visualized through the GROOVE approach, the presence of moderate to very high overlap in specific clusters indicates that some findings may rely on partially redundant primary studies. This limits the extent to which conclusions can be interpreted as independent confirmations across reviews and may lead to an overestimation of consistency in certain clinical domains, particularly stroke rehabilitation.

Moreover, the absence of a standardized and universally accepted definition of what constitutes a VR-based intervention further contributed to variability across studies, creating barriers to replication and clinical translation.

With regard to the search strategy, the decision not to extend the search to additional databases was based on preliminary exploratory checks showing a high degree of overlap with the evidence retrieved from the databases already used. In particular, Embase was not included because a targeted preliminary assessment indicated substantial citation redundancy with other consulted sources and, given its predominantly pharmacological orientation, its expected added value for this topic was limited.

Only a small proportion of the included reviews (2 out of 17) formally applied the GRADE approach. This substantially limits the ability of the umbrella review to draw firm conclusions regarding the overall certainty of evidence for VR-based interventions across chronic conditions.

Finally, the review protocol was not prospectively registered in an international database, which may be regarded as a methodological limitation.

## 5. Conclusions

This umbrella review synthesizes the available evidence on the use of virtual reality interventions in chronic conditions, highlighting their potential benefits across physical, respiratory, cognitive, and psychological domains, particularly in post-acute rehabilitation settings. However, the strength and consistency of evidence vary considerably across conditions and outcome domains.

While findings suggest that VR-based interventions may represent a promising complementary approach, especially for stroke and COPD rehabilitation, the interpretation of effects should account for substantial heterogeneity in interventions, outcomes, and study designs, as well as for the presence of overlapping primary evidence in certain pathology-specific clusters.

Consequently, current evidence supports cautious and context-specific integration of VR into chronic care rather than broad generalization. Future research should prioritize standardized intervention protocols, rigorous assessment of long-term outcomes, transparent reporting of implementation processes, and expansion to underrepresented populations and care phases, including acute settings. Addressing issues of equity, accessibility, and ethical implications will be essential before VR can be confidently recommended as a scalable component of chronic disease management.

## Figures and Tables

**Table 1:** PICO Framework.

PICO Element	Description
Population (P)	People living with chronic conditions
Intervention (I)	Use of virtual reality to support the management of chronic conditions
Comparison (C)	Any comparator reported in the included reviews (e.g., usual care, alternative therapy, or no intervention)
Outcome (O)	All outcomes measured in the included reviews, including symptoms, functional outcomes, quality of life, adherence, well-being, and any other outcomes related to the management of chronic conditions

**Table 2:** Quality appraisal of the included reviews.

Author(s)	Q1	Q2	Q3	Q4	Q5	Q6	Q7	Q8	Q9	Q10	Q11
Tuena et al. (2023) [30]	YES	YES	YES	YES	YES	YES	YES	YES	YES	YES	YES
Zhang et al. (2021) [8]	NO	YES	YES	YES	YES	YES	YES	YES	YES	YES	YES
Lee et al. (2019) [33]	NO	YES	YES	YES	YES	NO	YES	YES	YES	YES	YES
Patsaki et al. (2023) [28]	NO	YES	YES	YES	YES	YES	YES	YES	NO	YES	YES
Obrero-Gaitán et al. (2024) [29]	NO	NO	YES	YES	YES	YES	YES	YES	YES	YES	YES
Chai et al. (2023) [23]	NO	NO	YES	YES	YES	NO	YES	YES	YES	YES	YES
Kantha et al. (2023) [32]	YES	YES	YES	YES	YES	YES	YES	YES	YES	NO	NO
Zhong et al. (2021) [25]	NO	YES	YES	YES	YES	YES	YES	YES	Unclear	YES	YES
Alhwoaimel et al. (2024) [22]	NO	YES	YES	YES	YES	YES	NO	YES	NO	YES	YES
Shen et al. (2023) [24]	NO	YES	YES	YES	YES	YES	YES	YES	YES	YES	YES
Liu et al. (2023) [35]	NO	YES	YES	YES	YES	YES	YES	YES	NO	YES	YES
Alhusamiah et al. (2024) [6]	NO	YES	YES	YES	YES	YES	YES	YES	YES	YES	YES
Iruthayarajah et al. (2016) [27]	YES	YES	YES	YES	YES	Unclear	YES	YES	YES	Unclear	NO
McGhee et al. (2024) [31]	YES	YES	YES	YES	YES	Unclear	YES	YES	YES	YES	YES
Nascimento et al. (2021) [26]	YES	YES	YES	YES	YES	YES	YES	YES	YES	YES	YES
Rodrigues-Baroni et al. (2014) [21]	YES	YES	YES	YES	YES	YES	YES	YES	NO	NO	NO
Høeg et al. (2021) [34]	YES	YES	YES	YES	NO	NO	YES	YES	YES	YES	YES

**Table 3:** CCA matrix showing overlap of primary studies across included systematic reviews (GROOVE classification).

	Rev1	Rev2	Rev3	Rev4	Rev5	Rev6	Rev7	Rev8	Rev9	Rev10	Rev11	Rev12	Rev13	Rev14	Rev15	Rev16	Rev17
Rev1	–	0.57	0.42	0	0	0	0	0	0	0	0.09	0	0	0	0	0	0
Rev2	0.57	–	0.33	0	0	0	0	0	0	0	0.22	0	0	0	0	0	0
Rev3	0.42	0.33	–	0.037	0	0	0	0	0	0	0.55	0	0	0	0	0	0
Rev4	0	0	0.037	–	0	0	0	0	0	0	0.05	0	0	0	0	0	0
Rev5	0	0	0	0	–	0	0	0	0	0	0	0	0	0	0	0	0
Rev6	0	0	0	0	0	–	0	0	0	0	0	0	13.5	8.3	12.0	9.5	17.6
Rev7	0	0	0	0	0	0	–	0	0	0	0	0	4.8	0	0	0	0
Rev8	0	0	0	0	0	0	0	–	0	0	0	0	0	0	0	0	0
Rev9	0	0	0	0	0	0	0	0	–	0	0	0	15.8	12.0	7.1	8.7	14.7
Rev10	0	0	0	0	0	0	0	0	0	–	0	0	3.3	0	0	0	6.7
Rev11	0.09	0.22	0.55	0.05	0	0	0	0	0	0	–	0	13.3	11.1	5.3	6.3	15.4
Rev12	0	0	0	0	0	0	0	0	0	0	0	–	9.5	5.9	0	0	7.7
Rev13	0	0	0	0	0	13.5	4.8	0	15.8	3.3	13.3	9.5	–	23.5	11.5	12.0	13.9
Rev14	0	0	0	0	0	8.3	0	0	12.0	0	11.1	5.9	23.5	–	22.2	15.0	21.4
Rev15	0	0	0	0	0	12.0	0	0	7.1	0	5.3	0	11.5	22.2	–	0	8.3
Rev16	0	0	0	0	0	9.5	0	0	8.7	0	6.3	0	12.0	15.0	0	–	15.0
Rev17	0	0	0	0	0	17.6	0	0	14.7	6.7	15.4	7.7	13.9	21.4	8.3	15.0	–

Legend: Green = 0; Yellow = 0–5% (slight overlap); Orange = 6–10% (moderate overlap); Light Brown = 11–15% (high overlap); Dark Brown = >15% (very high overlap). Rev 1 = Patsaki et al. (2023) [M22.1] [28]; Rev 2 = Obrero-Gaitán et al. (2024) [29]; Rev 3 = Chai et al. (2023) [23]; Rev 4 = Zhong et al. (2021) [25]; Rev 5 = Nascimento et al. (2021) [26]; Rev 6 = Shen et al. (2023) [24]; Rev 7 = Alhwoaimel et al. (2024) [22]; Rev 8 = McGhee et al. (2024) [31]; Rev 9 = Zhang et al. (2021) [8]; Rev 10 = Alhusamiah et al. (2024) [6]; Rev 11 = Liu et al. (2023) [35]; Rev 12 = Kantha et al. (2023) [32]; Rev 13 = Lee et al. (2019) [33]; Rev 14 = Høeg et al. (2021) [34]; Rev 15 = Tuena et al. [30]; Rev 16 = Rodrigues-Baroni et al. (2014) [21]; Rev 17 = Iruthayarajah et al. [27].

**Table 4:** GRADE ratings.

Outcome	Comparison	Effect Size (SMD)	Certainty of Evidence
Functional capacity	Overall	0.40 (0.07–0.71)	Low
Functional capacity	VRBT vs. PR	0.51 (0.24–0.78)	Very low
Functional capacity	VRBT + PR vs. PR	0.38 (0.07–0.70)	Low
Pulmonary function	VRBT vs. PR	0.33 (0.01–0.65)	Very low
Functional mobility	Overall	0.77 (0.50–1.10)	Low
Functional mobility	VRBT + PR vs. PR	0.80 (0.45–1.45)	Very low
Global cognition (MoCA)	Intervention vs. control	0.42 (0.04–0.79)	Moderate
Global cognition (MMSE)	Intervention vs. control	0.09 (−0.26–0.44)	Moderate
Delayed memory	Intervention vs. control	0.31 (−0.05–0.68)	Moderate
Immediate memory	Intervention vs. control	0.00 (−0.28–0.29)	Moderate
Executive function (Trail A)	Intervention vs. control	−0.58 (−0.80 to −0.35)	Moderate
Executive function (Trail B)	Intervention vs. control	−0.07 (−0.31–0.18)	Moderate
Attention (DSF)	Intervention vs. control	−0.24 (−0.75–0.26)	Moderate
Attention (DSB)	Intervention vs. control	0.03 (−0.47–0.53)	Moderate
Instrumental activities of daily living (IADL)	Intervention vs. control	0.40 (−0.14–0.94)	Moderate

Legend: Effect sizes are reported as standardized mean differences (SMD). Certainty of evidence was rated according to the GRADE approach and classified as high, moderate, low, or very low. VRBT = virtual reality–based therapy; PR = pulmonary rehabilitation; DSF = Digit Span Forward; DSB = Digit Span Backward; IADL = instrumental activities of daily living; MoCA = Montreal Cognitive Assessment; MMSE = Mini-Mental State Examination.

## Data Availability

Data of Extraction table will be provided by the corresponding author on a specific request.

## Supplementary Materials

The following supporting information can be downloaded at: https://www.mdpi.com/article/10.3390/nursrep16020057/s1, Table S1: Keywords and Search strategies.

## Public Involvement Statement

No public involvement in any aspect of this research.

## Guidelines and Standards Statement

PRISMA Statement: Defining standard protocol items for clinical trial [13].
