# Supplementary material for: Virtual Reality in Chronic Conditions: An Umbrella Review
Source: Nurs Rep. 2026 Feb 10;16(2):57. doi: 10.3390/nursrep16020057 (PMC12943612; doi:10.3390/nursrep16020057)
Supplement: Supplementary file 1 [file nursrep-16-00057-s001.zip › nursrep-4120271-supplementary.pdf]

Table S1. Keywords and Search Strategies.

**MEDLINE via PubMed**
(((((((((((chronic illness[MeSH Terms]) OR (chronic disease[MeSH Terms])) OR (chronic illnesses[MeSH Terms])) OR (chronic diseases[MeSH Terms])) OR ("chronic illness"[Title/Abstract])) OR ("chronic disease"[Title/Abstract])) OR ("chronic illnesses"[Title/Abstract])) OR ("chronic diseases"[Title/Abstract])) AND (extended reality[MeSH Terms])) OR (virtual reality[MeSH Terms])) OR ("extended reality"[Title/Abstract])) OR ("virtual reality"[Title/Abstract])) Filters: Meta-Analysis, Systematic Review
**CINAHL**
((MH "Chronic Disease") OR ((TI "chronic disease" OR AB "chronic disease")) OR ((TI "chronic illness" OR AB "chronic illness")) OR ((TI "chronic diseases" OR AB "chronic diseases")) OR ((TI "chronic illnesses" OR AB "chronic illnesses"))) AND ((MH "Virtual Reality+") OR ((TI "virtual reality" OR AB "virtual reality")) OR ((TI "extended reality" OR AB "extended reality"))) Limiters: Systematic Review
**Scopus**
TITLE-ABS-KEY ("chronic illness" OR "chronic disease" OR "chronic illnesses" OR "chronic diseases") AND TITLE-ABS-KEY ("extended reality" OR "virtual reality") AND (LIMIT-TO (DOCTYPE , "re"))
**Cochrane Database**
("chronic illness" OR "chronic disease" OR "chronic illnesses" OR "chronic diseases") AND ("extended reality" OR "virtual reality")
**Web of Science**
("chronic illness" OR "chronic disease" OR "chronic illnesses" OR "chronic diseases") AND ("extended reality" OR "virtual reality") AND ("systematic review" OR "meta-analysis" OR "meta analysis")
